# Persistent Infections in Tick Cell Lines: The Role of Viral-Derived DNA Forms in Hazara Virus Replication and Cellular Survival

**DOI:** 10.3390/v17050591

**Published:** 2025-04-22

**Authors:** Eva Dias, Filipe Tomaz, Silvia Fabi, Cristiano Salata, Ana Domingos, Gonçalo Seixas

**Affiliations:** 1 Global Health and Tropical Medicine (GHTM), Associate Laboratory in Translation and Innovation Towards Global Health, LA-REAL, Instituto de Higiene e Medicina Tropical (IHMT), Universidade Nova de Lisboa, UNL, Rua da Junqueira 100, 1349-008 Lisboa, Portugal; eva.dias@gimm.pt (E.D.); tomazfilipe88@gmail.com (F.T.); adomingos@ihmt.unl.pt (A.D.); 2 Gulbenkian Institute for Molecular Medicine (GIMM), Avenida Professor Egas Moniz, 1649-028 Lisboa, Portugal; 3 Department of Molecular Medicine, University of Padova, Via A. Gabelli 63, 35121 Padua, Italy; silvia.fabi@studenti.unipd.it (S.F.); cristiano.salata@unipd.it (C.S.)

**Keywords:** Crimean–Congo hemorrhagic fever virus, Hazara virus, nairoviruses, tick cell lines, viral derived DNA forms, tick-borne viruses, *H. lusitanicum*, bunyavirus, reverse transcriptase

## Abstract

Crimean–Congo hemorrhagic fever virus (CCHFV) causes severe or fatal infections in humans and is geographically widespread. The virus has coevolved with its tick vectors, establishing persistent infections critical to its transmission. This study explored the mechanisms underpinning these persistent infections, using tick cell lines and the Hazara virus (HAZV) as a biosafety level 2 (BSL-2) model for CCHFV. Initially, an RT-qPCR protocol was developed to detect HAZV in tick cells. The study then focused on the production of virus-derived DNA (vDNAs) by tick cells as a defensive response to infection. These vDNAs regulate viral particle production, enabling tick cells to maintain viability and establish persistent infections. The experiments characterized vDNAs production, viral titers, and subcellular localization, and they examined the effect of the reverse transcriptase inhibitor azidothymidine triphosphate (AZT). The results showed that all tested tick cell lines supported HAZV replication, achieving persistent infections without cytopathic effects. vDNAs was detected in both the cytoplasm and nucleus, and its formation was dependent on HAZV infection. Importantly, vDNAs presence was linked to infection persistence; cells treated with AZT exhibited a marked reduction in vDNAs production and an associated increase in viral particle production, which correlated with higher cell death. These findings underscore the critical role of vDNAs in balancing viral replication and promoting long-term cell survival in tick cells, highlighting their importance in the coevolution of tick-borne viruses and their vectors.

## 1. Introduction

Crimean–Congo hemorrhagic fever (CCHF) is caused by the Crimean–Congo hemorrhagic fever virus, classified as *Orthonairovirus haemorragiae* (CCHFV), a tick-borne virus primarily transmitted by ticks of the *Hyalomma* genus. The disease has a mortality rate of up to 40% [1]. In recent decades, the geographic range of the CCHFV’s tick vectors has expanded significantly, leading to an increase in the distribution of the disease [2,3]. Understanding the molecular mechanisms underlying viral infection and transmission is crucial for developing strategies to combat this pathology. To study these interactions, the Hazara virus, classified as *Orthonairovirus hazaraense* (HAZV), is commonly used as a surrogate for CCHFV due to its genetic, serological, and molecular similarities, offering a safer and more accessible experimental model for investigating CCHFV [4,5,6,7,8,9]. This approach enables experimental procedures that closely mimic CCHFV infections while minimizing biosafety risks [10].

As an arbovirus, CCHFV is part of an ecological and polyphyletic group of viruses transmitted by arthropods. For an arthropod to act as a vector, it must support viral replication and enable disease transmission [11,12,13]. The biological transmission of CCHFV depends on several factors, including the virus’s ability to replicate in arthropod cells, vector competence, host contact, and host susceptibility. To date, virus–host interactions are poorly characterized, including the mechanisms responsible for establishing persistent infections in tick cells [14,15,16].

Persistent infections of ticks play a critical role in virus transmission, as infected ticks can transmit the virus throughout their lifetime to other ticks or vertebrate hosts. Once a tick becomes infected, the virus can persist through transstadial transmission (across developmental stages) and transovarial transmission (from infected females to their offspring), making ticks the reservoir of CCHFV. Viral DNA-derived forms (vDNAs) have emerged as key players in establishing these persistent infections. Initially observed in mosquitoes and more recently in ticks, vDNAs are thought to play a significant role in the immune responses of arthropods [16,17,18,19,20].

These vDNAs may exist in two forms: integrated into the host genome or as episomal molecules. Both forms likely contribute to antiviral defense by generating small interfering RNAs (siRNAs) that regulate viral replication [21,22,23,24]. Initially reported in mosquitoes, the presence of vDNAs in ticks was only recently observed [25]. Nairoviruses, including CCHFV, do not encode reverse transcriptase in their genomes. Instead, it is hypothesized that this activity originates from host cell retrotransposons and retroviral sequences. Reverse-transcriptase-like proteins have been identified in tick species such as *Amblyomma americanum* and *Ixodes scapularis* [25], and many retrotransposons were recently reported in *Hyalomma anatolicum* tick genomes [26]. The formation of vDNAs is thought to involve template-switching events, where reverse transcriptase shifts from retro-element templates to viral RNA, resulting in the creation of vDNAs-retrotransposon chimeric molecules [23,24,27,28].

Although rare, the integration of viral sequences into the genome is believed to play a critical role in virus–vector coevolution. Notably, sequences resembling bunyaviruses and orthomyxoviruses have been found in the *I. scapularis* genome, indicating that these viruses can produce vDNAs in ticks and occasionally integrate them into germline cells [28].

Recent studies show that vDNAs are produced in tick cells during infections with both HAZV and CCHFV, and they appear to be a part of a complex defense mechanism in tick cells, critical for cellular survival [16].

The primary objective of this research is to investigate the mechanisms underlying the establishment of persistent infections in Hyalomma cell lines infected with HAZV, focusing specifically on the role of vDNAs. An RT-qPCR protocol was established to detect HAZV in tick cells and monitor viral presence and replication. Additionally, this study examined the dynamics of vDNAs production, their cellular localization, and their influence on viral replication and cell survival. The findings aim to enhance our understanding of the molecular interactions between HAZV and tick cells, shedding light on the processes involved in the establishment of persistent infections and bridging existing knowledge gaps in this field.

## 2. Materials and Methods

### 2.1. Design and Optimization of a New Molecular Method for Detection and Quantification of HAZV Genomic Material

A TaqMan-based RT-qPCR assay was developed to assess the dynamic infection of HAZV in mammalian and in tick cell lines over time. The assay was designed to ensure high specificity and sensitivity for detecting HAZV genomic material. Primers and probes were designed using the Primer3 software tool (version 4.1.0), targeting the S segment of the HAZV genome (isolate JC280). The primers and probe were synthesized by SATB Vida (Caparica, Portugal) and their sequences are presented in Table 1.

For optimization, a range of primer concentrations (100–600 nM) and annealing temperatures (54–60 °C) were tested using SYBR Green dye as the fluorophore. Subsequently, probe concentrations (50–250 nM) were evaluated in combination with fixed primer concentrations (250 nM and 400 nM) to identify the optimal assay conditions. To quantify viral genomic material, a synthetic standard (gBlocks) of 496 nucleotides was designed to represent the target sequence from IDT (Coralville, IA, USA). This standard was used to generate standard curves for precise quantification and served as a positive control in RT-qPCR reactions [29].

### 2.2. Cell Lines

#### 2.2.1. Tick Cells

The *Hyalomma anatolicum* (HAE/CTVM9) [30], *Ixodes scapularis* (ISE6) [31], and *Hyalomma lusitanicum* (HLE/LULS42) [32] cell lines were obtained from the Tick Cell Biobank through a collaboration with Dr. Lesley Bell-Sakyi (University of Liverpool). HAE/CTVM9 cells were cultured in a 1:1 mixture of Leibovitz’s L-15 Medium (L15) and Minimum Essential Medium (MEM) with Hank’s salts. The medium was supplemented with 10% Tryptose Phosphate Broth (TPB), 20% Fetal Bovine Serum (FBS), 2 mM L-glutamine, and Pen/Strep (10,000 U/mL penicillin and 10,000 µg/mL streptomycin). ISE6 cells were cultured in a 3:1 mixture of L15B and Milli-Q water, with L15B supplemented with 10% TPB, 5% FBS, 0.1% lipoprotein, 2 mM L-glutamine, and Pen/Strep (10,000 U/mL penicillin and 10,000 µg/mL streptomycin). HLE/LULS42 cells were cultured in a 1:1 mixture of L15 and L15B medium. L15 medium was supplemented with 10% TPB, 20% FBS, 2 mM L-glutamine, and Pen/Strep (10,000 U/mL penicillin and 10,000 µg/mL streptomycin). L15B medium was supplemented with 10% TPB, 5% FBS, 2 mM L-glutamine, 0.1% lipoprotein, and Pen/Strep (10,000 U/mL penicillin and 10,000 µg/mL streptomycin). All tick cell lines were grown in 2 mL Nunc™ tubes and maintained in CO_2_-free incubators. ISE6 and HAE/CTVM9 cells were incubated at 32 °C, while HLE/LULS42 cells were incubated at 28 °C. The cells were split every 3–4 weeks or whenever the medium required changing (approximately once a week). The preparation of all culture media for tick cell growth and maintenance followed the guidelines of Munderloh and Kurtti [33].

#### 2.2.2. Mammalian Cells

The SW13 cell line, derived from human adrenal cortex carcinoma (ATCC CCL-105™), was used for the production and quantification of HAZV. SW13 cells were cultured in Leibovitz’s L-15 Medium, supplemented with 10% Fetal Bovine Serum (FBS), 2 mM L-glutamine, and Pen/Strep (10,000 U/mL penicillin and 10,000 µg/mL streptomycin). The cells were maintained in sealed flasks in a CO_2_-free incubator at 37 °C. The cells were passaged every 3–4 days using 0.25% Trypsin-EDTA. This ensured optimal growth and prevented over-confluency, facilitating their use in experiments.

### 2.3. HAZV Virus

#### 2.3.1. Production

The virus was produced by seeding 5 million SW13 cells in T75 flasks and inoculating them with the viral stock at a multiplicity of infection (MOI) of 0.1. After incubation at 37 °C for 1 h, the viral inoculum was removed, and the flasks were supplemented with Leibovitz’s L-15 Medium (L15) containing 10% Fetal Bovine Serum (FBS) and 2 mM L-glutamine. The virus was harvested 48 h post-infection and stored at −80 °C until further use.

#### 2.3.2. Quantification

SW13 cells were seeded in 12-well plates and incubated until reaching confluency. Once confluent, the medium was removed, and HAZV was added in duplicate after serial dilutions (10-fold) in Leibovitz’s L-15 Medium (L15). For the negative control (MOCK), only L15 medium was used. The plates were incubated for 1 h at the same temperature of their growth, allowing viral adsorption. Following this incubation, the viral inoculum was removed, and an overlay was applied by mixing equal parts of 2% carboxymethylcellulose (CMC) (CMC sodium salt, low viscosity) from Merck (E. Merck KG, Darmstadt, Germany) and L15 2X, supplemented with 5% FBS and 2 mM L-glutamine.

The plates were incubated for 6 days at 37 °C without agitation. After this period, the cells were fixed with 20% formaldehyde and stained with 0.1% crystal violet. To fix the cells, 20% formaldehyde in phosphate-buffered saline (PBS) from Sigma-Aldrich (Sigma-Aldrich, St. Louis, MO, USA) was added to each well, and the mixture was incubated for 1 h. The plates were then submerged in water and washed to remove residual formaldehyde. Next, a 0.1% crystal violet solution (diluted in 50% ethanol) was added until the surface of each well was fully covered, and the mixture was incubated for 15–20 min. After staining, the plates were washed again to remove excess crystal violet by submerging them in water.

The viral plaques were subsequently counted, and the viral titer was calculated as 6.39 × 10^6^ PFU/mL using Equation (1).

(1) $$PFU/\mathrm{mL}=\frac{Average\;number\;of\;plaques}{inoculum\;volume\left(\mathrm{mL}\right)*Total\;dilution\;factor}$$

Equation (1)—Viral titer calculation (PFU/mL).

### 2.4. Infection of Tick Cell Lines

Tick cells were seeded into individual tubes and incubated for 48 h at the temperature appropriate for their maintenance and growth. After 2 days, the medium was removed, and 1 mL of viral inoculum, containing the appropriate concentration of virus and the respective medium for the cell line, was added to achieve the desired multiplicity of infection (MOI) (0.1, 1, and 5). For the negative control groups (MOCK), only medium was added during this step. All tubes were incubated for 1 h at 37 °C to allow for viral adsorption. After this incubation period, the inoculum was removed, and the cells were washed with phosphate-buffered saline (PBS). Fresh medium was then added, and the cultures were incubated at the temperature appropriate for their maintenance and growth until collection at specific time points.

To assess the kinetics of HAZV infection in ISE6, HAE/CTVM9, and HLE/LULS42 tick cell lines, 200 µL of supernatant medium was collected from the cultures at specific time points. An equal volume of fresh medium was added to replace the collected supernatant in the tubes. The collected supernatants were stored at −80 °C until further processing. RNA extraction was performed on 50 µL of the supernatant from each time point for molecular quantification of the viral genome.

For the detection of vDNAs in HLE/LULS42 cells, both the cells and medium from the culture tubes were collected at specific time points. The tube contents were harvested and centrifuged at 10,000× *g* for 5 min to separate the pellet (cells) and supernatant (medium). Both phases were stored separately at −80 °C. The pellet was used for DNA extraction, while the supernatant was processed for RNA extraction.

### 2.5. Cell Viability Assay Using Trypan Blue

The trypan blue dye exclusion assay was employed to distinguish between viable and non-viable cells during cell counting. At the time of cell collection, all cells were detached from the tube surface, suspended in the medium, and mixed to ensure a uniform cell suspension. A 20 µL aliquot of the cell suspension was mixed with 180 µL of trypan blue solution in a 1:10 ratio. After 1–2 min of incubation at room temperature, the mixture was carefully loaded into a counting chamber (hemocytometer).

Cells were then counted under an optical microscope. Viable cells excluded the dye and appeared unstained, while non-viable cells absorbed the dye and appeared blue. The viability percentage was calculated based on the number of unstained cells relative to the total number of cells.

### 2.6. RNA Extraction

Two distinct methods were employed for RNA extraction to address specific experimental objectives: one for quantifying viral progeny from the extracellular medium and another for assessing intracellular viral genome synthesis. Both methods ensured the extraction of high-quality RNA suitable for downstream applications such as molecular quantification and analysis.

RNA extraction from the supernatant of infected tick cell cultures was performed using NZYol reagent (NZYtech, Lisboa, Portugal), following the manufacturer’s instructions.

To evaluate the synthesis of the viral genome inside tick cells, RNA extraction was also performed using the DNA, RNA & Protein Extraction Kit from Bio Basic (Bio Basic Inc., Markham, ON, Canada) according to the manufacturer’s instructions. The final RNA product was stored at −80 °C for further analysis. Both extraction methods were essential for obtaining high-quality RNA from different biological compartments, ensuring comprehensive viral RNA analysis.

### 2.7. DNA Extraction

To evaluate the formation of vDNAs, DNA extraction from tick cells was performed using two complementary methods: one for extracting total cellular DNA and another for assessing the subcellular localization of vDNAs by separating nuclear and cytoplasmic fractions. Both methods ensured the isolation of high-quality DNA for further molecular analysis.

Total DNA extraction was performed using the DNA, RNA & Protein Extraction Kit (Bio Basic), following the manufacturer’s instructions. The extracted DNA was stored at −20 °C for downstream applications.

To determine the subcellular localization of vDNAs, an adapted DNA extraction method was used, based on a protocol described for *Aedes aegypti* [17]. This procedure enabled the separation of nuclear and cytoplasmic DNA fractions. The protocol began by washing a pellet containing 1 million tick cells twice with sterile PBS. The cells were then lysed by adding 100 µL of Lysis Buffer (100 mM Tris-HCl, 1 mM EDTA, 0.65% Nonidet P40, and 150 mM NaCl), followed by vortex homogenization. The lysate was centrifuged at 1500× *g* for 5 min at 4 °C, and the supernatant was transferred to a new tube on ice, representing the cytoplasmic fraction. The pellet, containing the nuclear fraction, was resuspended in an additional 100 µL of Lysis Buffer.

To both the nuclear and cytoplasmic fractions, 20 µL of Proteinase K (20 mg/mL) was added, and the samples were incubated in a thermocycler for 2 h at 52 °C, followed by 10 min at 96 °C to inactivate enzymes. The final extracted DNA was stored at −20 °C until further processing.

This combined approach allowed for a comprehensive analysis of vDNAs, providing insights into their distribution within tick cells and their potential role in persistent viral infections.

### 2.8. Reverse Transcription Quantitative PCR (RT-qPCR)

The assessment of the genomic material in the samples was conducted via One-Step TaqMan probe RT-qPCR. All the samples were analyzed in duplicate. Every reaction consisted of a 10 µL total volume of which 5 µL were of iTaq Universal Probes One-Step Kit Master Mix from Bio-Rad (Hercules, CA, USA), 250 nM of each primer, 50 nM of probe, and 3 µL of RNA to be analyzed. Nuclease-free water was added to a final reaction volume of 10 µL. The cycling conditions used were as indicated in Table 2, and the fluorescence data were collected after each 60 °C annealing step. The standard curve was generated using 10-fold serial dilutions of gBlocks as a template to evaluate the kinetics of infection in tick cell lines (HAE/CTVM9, ISE6, and HLE/LULS42), with results expressed in copies/mL.

For the quantification of the viral titer in the vDNA-related assay, a different method was applied. A calibration curve was generated by correlating the quantitation cycle (Cq) values with previously determined PFU/mL from a plaque assay, allowing the results to be expressed in PFU/mL.

The analysis of the MOCK uninfected samples (the negative infection control) was also conducted and was treated the same way as other samples. A negative reaction control, with DEPC-treated water instead of the RNA template but containing all other components of the reaction mix, was used to test for possible contamination.

### 2.9. Conventional PCR for the Detection of vDNAs

For the detection of vDNAs forms, 9 pairs of primers developed to amplify overlapping sequences ranging from 152 to 237 base pairs (bp) and covering the S segment of HAZV were used [16]. 

The reaction mix was prepared by combining 5 µL of NZYTaq II 2× Green Master Mix, 0.4 µL of each forward and reverse primer to achieve a final concentration of 200 nM, and nuclease-free water to bring the total volume to 20 µL. The volume of the sample added varied depending on the DNA extraction method used. Specifically, 2.5 µL of the sample was extracted using the commercial kit and 5 µL of the sample when extracted from cytoplasmic/nuclear fractions. All the samples were processed with the cycling conditions presented in Table 3 using the T100 thermocycler from Bio-Rad (Hercules, CA, USA).

### 2.10. Electrophoresis

Electrophoresis was performed to verify the specificity of the PCR reaction and confirm the amplification of the expected products. Several agarose gels were prepared for this purpose.

Agarose gels were prepared using a 2% agarose concentration in Tris-Borate-EDTA Buffer (TBE). This mixture was heated until completely dissolved. Once cooled slightly, GreenSafe Premium (3 µL/100 mL of gel) was added to facilitate DNA visualization. After gel polymerization, 15 µL of each sample was loaded into individual wells, along with 2 µL of the molecular weight marker (NZYDNA Ladder V, Nzytech (Nzytech, Lda, Lisbon, Portugal). Electrophoresis was conducted for 35 min at 140 V.

Following electrophoresis, the gels were visualized and photographed using the ChemiDoc MP Imaging System (Bio-Rad) to document the results.

### 2.11. Treatment of Tick Cells with AZT

To evaluate the impact of reverse transcriptase inhibition on vDNAs formation, viral progeny, and cell survival, an assay was conducted using AZT, a known reverse transcriptase inhibitor.

The AZT drug (Sigma) was dissolved in DMSO to obtain a stock concentration of 5M and stored in small aliquots at −20 °C. Treatment was performed by removing 1.2 mL of medium from each tube and replacing it with the same volume of fresh medium containing AZT at a final concentration of 5 mM.

For this assay, persistently infected cells (more than 2 months post-infection) and uninfected control cells were seeded at 2 million cells per tube. After an incubation period of two days, AZT (5 mM) was added to the experimental groups. Following 72 h of treatment, the cells were collected for further analysis.

Two negative control groups were included in the experiment. One consisted of infected cells that were not subjected to any treatment and received only fresh medium. The second control group received the same volume of DMSO used to dissolve AZT to rule out any solvent effects.

All groups were processed identically. After collection, all groups underwent cell counting, and infected groups were further analyzed for cell viability, vDNAs formation, viral progeny quantification, and viral genome replication. This experimental setup ensured a comprehensive evaluation of the role of vDNAs formation in persistent viral infections and the effects of reverse transcriptase inhibition on tick cell survival and viral dynamics.

### 2.12. Data Analysis

All statistical analyses and graph generation were performed using GraphPad Prism software (version is 10.4.2). Statistical tests were selected based on the nature of the data and the experimental design.

A paired *t*-test was used to compare the number of live cells per mL between infected and uninfected groups over a 10-day post-infection period. A two-way ANOVA was applied to analyze vDNAs production and viral progeny dynamics over 60 days in infected tick cells at MOI 0.1 and 1. Additionally, a two-way ANOVA was used to compare the number of live cells in infected and uninfected groups treated with AZT, DMSO, and MOCK, with Tukey’s test used for multiple comparisons between the groups.

For infected cells treated with AZT, an ordinary one-way ANOVA was used to compare differences across the AZT, DMSO, and MOCK groups, specifically analyzing cell viability, vDNAs percentage, viral progeny, and viral replication, with Tukey’s test applied for multiple comparisons.

Finally, a *t*-test was conducted to compare the Log10 PFU per cell count between the AZT and MOCK groups. Differences were considered statistically significant at *p* < 0.05.

## 3. Results

### 3.1. Optimization of the Molecular Method to Detect and Quantify HAZV

The optimal primer and probe concentrations were determined by selecting the lowest concentration that maintained high efficiency while minimizing Cq values. Among the five concentrations tested, no significant differences in Cq values were observed, allowing the selection of the lowest effective concentration to optimize reaction efficiency. This optimized protocol ensures sensitive, specific, and efficient HAZV detection.

Probe optimization assays indicated no significant differences in reaction efficiency across the concentrations tested (Table 4). However, the lowest Cq value was achieved with a probe concentration of 100 nM combined with a primer concentration of 250 nM. This combination was selected for all subsequent reactions to maintain optimal sensitivity and specificity in detecting HAZV.

### 3.2. Tick Cell Line (HLE/LULS42, HAE/CTVM9, and ISE6) Infection with HAZV

**Figure 1:**
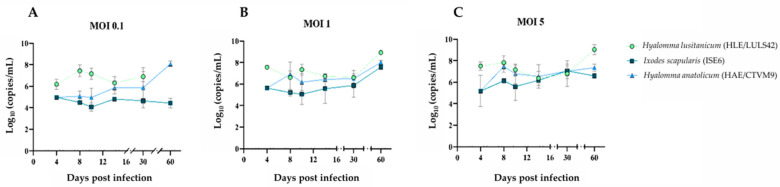
Evolution of HAZV infection in the tick cell lines. (**A**) represents cells infected at MOI 0.1, (**B**) corresponds to cells infected at MOI 1, and (**C**) shows cells infected at MOI 5. Error bars represent standard deviation (S.D.).

To evaluate the kinetics of infection, viral replication, and potential cytopathic effects in tick-derived cell lines, HLE/LULS42, HAE/CTVM9, and ISE6 cells were infected with HAZV. Following infection, supernatants from each cell line were collected at specific time points. RNA extraction and quantification by RT-qPCR were performed to assess the viral genomic material present in each sample, allowing for the analysis of infection dynamics over time; in the MOCK groups, no amplification was detected. The results are presented in Figure 1. Across all time points, HAZV viral titers in HLE/LULS42 cells were significantly higher than those observed in the ISE6 cell line at MOI 0.1 and 1. Notably, at 4 days post-infection (dpi), HAZV titers in HLE/LULS42 cells were substantially higher than those in HAE/CTVM9-infected cells at all three MOIs.

**Figure 2:**
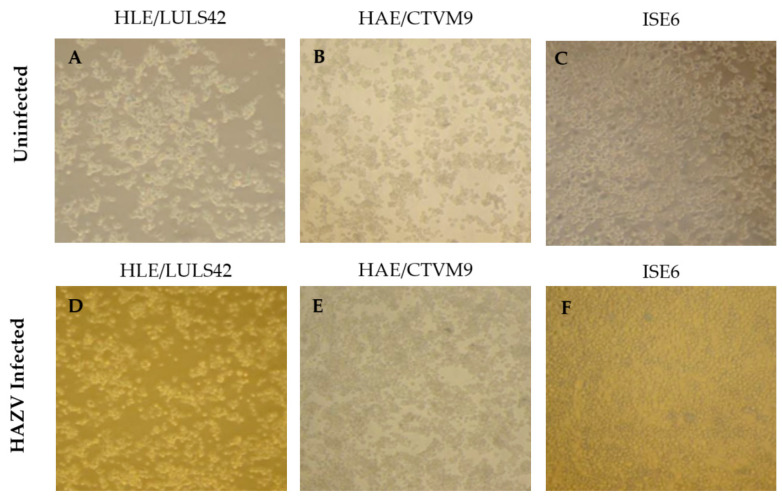
Comparison of morphology between infected and uninfected tick cells. The first row displays the morphology of uninfected tick cell lines: HLE/LULS42 (**A**), HAE/CTVM9 (**B**), and ISE6 (**C**) during their normal cell cycle. The second row shows the same cell lines post-infection with HAZV: HLE/LULS42 (**D**), HAE/CTVM9 (**E**), and ISE6 (**F**).

Additionally, no morphological changes were observed in the infected tick cell lines, as shown in Figure 2, suggesting that HAZV does not induce significant cytopathic effects in these cells.

Overall, results show that HAZV can infect all three tick cell lines, and the infection persists for 60 days without any significant cytopathic effect. In addition, HLE/LULS42 appears to be more susceptible to HAZV infection than HAE/CTVM9 and ISE6 cells.

### 3.3. The Development of a Persistent HAZV Infection in HLE/LULS42

As previously explored in this study, tick cells can establish persistent infections with HAZV. This persistence results from virus–vector interactions, enabling viral replication while preserving cell viability. In contrast to mammalian cells (SW-13), where HAZV infection leads to rapid cell death, tick cells remain viable despite ongoing viral replication [16].

**Figure 3:**
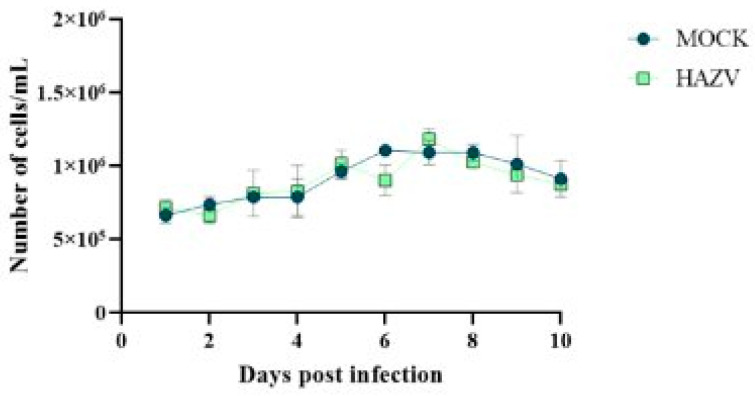
Cell viability in HAZV-infected and uninfected tick cells over 10 days. The graph illustrates the number of live cells per mL in HAZV-infected (MOI 0.1) and uninfected (MOCK) HLE/LULS42 cells over a 10-day post-infection period. Error bars represent standard deviation (S.D.).

Since *Hyalomma lusitanicum* is a relevant vector for CCHFV spread in the Iberian Peninsula, we focused our attention on HLE/LULS42 cells. Thus, an assay was conducted with two experimental groups: one infected at MOI 0.1 and another uninfected (MOCK) group serving as a control. This setup facilitated the comparison of cell viability between infected and uninfected groups over 10 days post-infection. The results of this experiment are presented in Figure 3. The results show no significant variation between the two groups, indicating that HAZV infection does not impact the viability of HLE/LULS42 tick cells.

### 3.4. Detection of HAZV-Derived DNA Forms in HLE/LULS42 Cells

To further investigate the mechanisms underlying persistent HAZV infections, this study focused on the formation of vDNAs in HAZV-infected HLE/LULS42 cells. The first step involved optimizing the PCR protocol, using viral cDNA as a template to ensure the accurate detection of vDNAs forms.

Following PCR optimization, HLE/LULS42 cells were infected with HAZV at MOIs of 0.1 and 1. At specific time points post-infection (3, 7, 15, 30, and 60 days), cells were harvested and subjected to DNA and RNA extraction. A negative control group (MOCK), consisting of uninfected cells, was included to assess baseline conditions.

**Figure 4:**
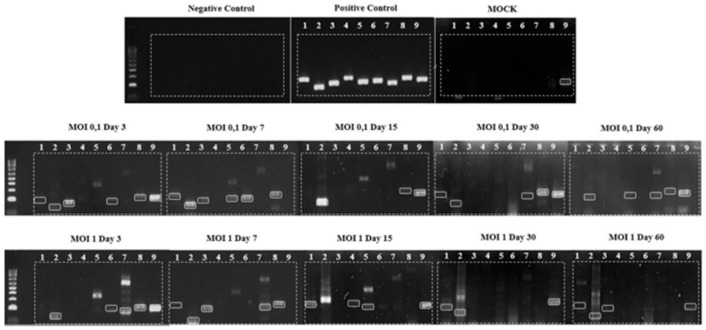
Detection of vDNAs in *HLE/LULS42* tick cells infected with HAZV. *H. lusitanicum* tick cells were infected with HAZV at MOIs of 0.1 and 1, and vDNAs detection was monitored over 60 days post-infection. PCR amplification was performed using nine primer pairs, targeting different regions of the viral genome. The upper panel displays control samples, the middle panel shows cells infected at MOI 0.1, and the lower panel presents cells infected at MOI 1. Highlighted bands indicate fragments of the expected size for each primer pair, confirming the presence of vDNAs in HAZV-infected cells.

DNA extraction was performed using the cell pellet, followed by conventional PCR utilizing nine primer pairs targeting overlapping sequences of the HAZV genome. The results, presented in Figure 4, demonstrate the presence of vDNAs in HLE/LULS42 cells infected with HAZV at MOIs of 0.1 and 1.

The figure also includes the control groups: the negative control, which confirms the absence of contamination; the positive control, using HAZV cDNA, validating the efficiency of the reaction; and the experimental control MOCK, where vDNAs detection was performed in uninfected cells. Amplification was observed only with the ninth primer pair in the MOCK group. Consequently, although the ninth primer pair is displayed in Figure 4, it was excluded from subsequent analyses.

These results indicate that HAZV infection actively induces the formation of vDNAs in infected HLE/LULS42 cells, consistent with findings from other *Hyalomma* cell lines (HAE/CTVM8 and HAE/CTVM9) [16]. This suggests that vDNAs generation is not specific to a particular tick cell line. Furthermore, this phenomenon appears to be independent of the MOI used and persists over time, reinforcing its potential role in establishing persistent infections.

In parallel with vDNAs detection, viral production in HAZV-infected HLE/LULS42 cells was quantified. RNA was extracted from the culture supernatant and analyzed using RT-qPCR to determine viral progeny levels, providing insights into viral replication dynamics over time.

**Figure 5:**
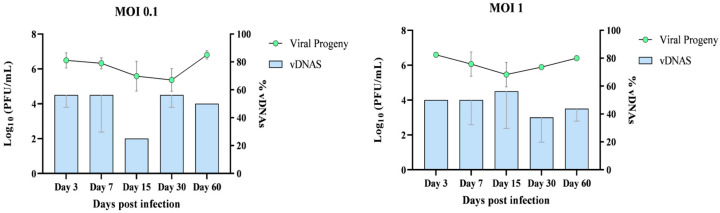
Comparation of viral titer (PFU/mL) and vDNAs formation in HLE/LULS42 cells infected with HAZV. HLE/LULS42 cells were infected with HAZV at MOIs of 0.1 and 1, and viral replication and vDNA formation were monitored over a 60-day infection period. Viral titers (PFU/mL) and vDNA levels were quantified at days 3, 7, 15, 30, and 60 post-infection. vDNAs levels are expressed as percentages relative to the maximum number of detectable amplicons (n = 8), set as 100% for each sample. Error bars represent standard deviation (S.D.).

This approach enabled a direct comparison between vDNAs production and viral progeny levels over a 60-day infection period. The summarized results of this analysis are presented in Figure 5.

Overall, the data show that vDNA was consistently detectable in persistently infected tick cell cultures, suggesting a potential role in long-term viral persistence.

#### Cellular Localization of vDNAs in the Nucleus and Cytoplasm

To determine the subcellular localization of vDNAs and gain further insights into the nature of these molecules, additional cell cultures were collected on days 7 and 15 post-infection. A specialized DNA extraction method [17] was employed to separate nuclear and cytoplasmic DNA.

**Figure 6:**
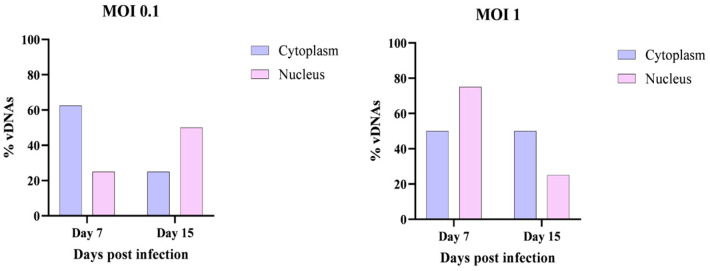
Detection of vDNAs in HLE/LULS42 tick cells infected with HAZV in the nucleus and cytoplasm. The percentages of vDNAs are expressed relative to the maximum number of detectable amplicons (n = 8), which was set at 100% for each sample.

The extracted DNA was subsequently analyzed by conventional PCR to detect vDNAs in each cellular compartment. The results, presented in Figure 6, confirm that vDNAs were detectable in both the nucleus and the cytoplasm at days 7 and 15 post-infection, regardless of the MOI used (0.1 or 1). These findings agree with the hypothesis that vDNAs are generated during retrotransposon activity and thus can be localized both in the cytoplasm and in the nucleus.

### 3.5. The Effect of vDNAs on the Establishment of Persistent Infections in HLE/LULS42

The AZT is a drug that inhibits RT activity. Consequently, administering AZT to infected tick cell cultures will inhibit the formation of vDNAs [16], allowing for the assessment of the impact of these molecules on the establishment of persistent infections.

**Figure 7:**
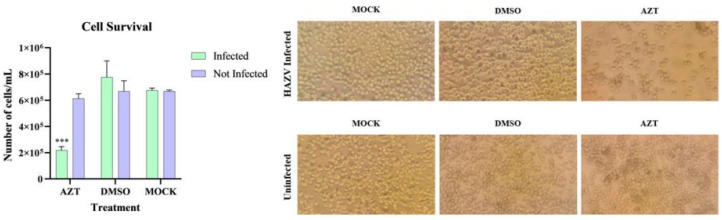
The effect of the drug AZT on infected and uninfected HLE/LULS42 tick cells. The only group affected was the infected one treated with AZT. ***, *p* < 0.001. Error bars represent standard deviation (S.D.).

To study this mechanism, cell lines that were previously infected (over two months post-infection with 0.1 MOI) and uninfected cells were treated with 5 mM AZT dissolved in DMSO and incubated for 72 h. Two negative control groups were included: one without AZT and another with DMSO. After incubation, all infected groups were processed for analysis, with the results shown in Figure 7.

When comparing the cell count per mL across the different groups, significant differences were observed only between the AZT-treated infected cells and those treated with DMSO or MOCK. No significant differences were found in the other groups, indicating that these compounds are not responsible for the decreased cell survival observed in infected.

**Figure 8:**
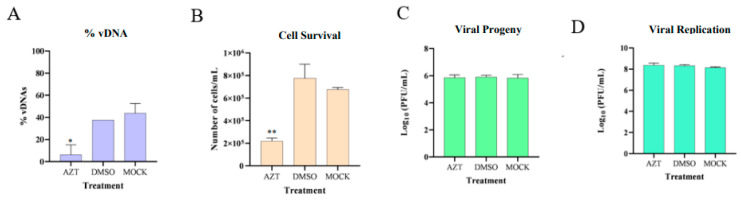
The effect of AZT treatment in HLE/LULS42 cells persistently infected with HAZV. Each group of cells (AZT, DMSO, and MOCK) received their specific treatment and were incubated for 72 h. Then, different assays were performed: (**A**) The percentage of detected vDNAs was evaluated by PCR. The percentages of vDNAs are expressed relative to the maximum number of detectable amplicons (n = 8), which was set at 100% for each sample. (**B**) The cell viability was determined by the trypan blue dye exclusion test. (**C**) The viral progeny present in cell supernatant was quantified by RT-qPCR. (**D**) The viral replication inside the cells was quantified by RT-qPCR. **, *p* < 0.01 and *, *p* < 0.05. Error bars represent standard deviation (S.D.).

The results of the infected groups experiments are presented in Figure 8, showing that the presence of AZT in infected tick cells inhibited the formation of vDNAs molecules, decreasing cell viability, while the viral progeny present in the supernatant and viral replication inside the cells remained constant. It is also apparent that there were no significant differences between the DMSO and MOCK groups, indicating that the DMSO solution itself does not have an impact and that the observed effects were exclusively caused by the added AZT.

**Figure 9:**
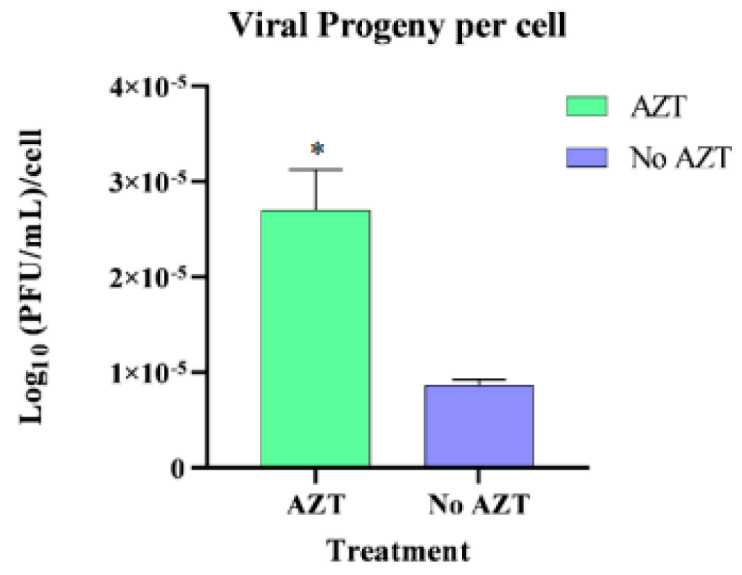
Comparison of viral particle production per cell in HLE/LULS42 cells persistently infected with HAZV following AZT treatment. AZT treatment led to a significant increase in the production of new viral particles per cell, suggesting that AZT may inhibit the formation of viral DNA, and this inhibition appeared to contribute to elevated viral production, which in turn can be associated with the observed cell death in this group. *, *p* < 0.05. Error bars represent standard deviation (S.D.).

These findings suggest that inhibiting vDNA formation leads to an increased release of viral progeny into the cell supernatant. Despite a reduction in cell numbers, the overall viral progeny levels remained constant across all groups. This indicates that surviving cells may produce more viral particles in the absence of vDNA, potentially linking the observed cell death to increased viral replication, presented in Figure 9.

## 4. Discussion

This study aimed to investigate the interactions between nairoviruses and their tick vectors, with a specific focus on elucidating the molecular mechanisms involved in the establishment of persistent infections in tick cell lines infected with HAZV. Initially, we characterized HAZV infection in tick cell lines, comparing its progression in different cell types and examining the role of vDNAs in HLE/LULS42 cells as a response to viral infection. HAZV was chosen as a surrogate model for the CCHFV, allowing the use of BSL2 containment conditions instead of the BSL4 facilities required for CCHFV manipulation. The use of HAZV as a model system has been proven to be an effective strategy for studying virus–vector interactions due to the genetic and serological similarities between the two viruses [5,6,7,8,9,16,34].

Our results demonstrated that HAZV successfully established a persistent infection across all tested tick cell lines (HLE/LULS42, ISE6, and HAE/CTVM9) [35,36]. Notably, HLE/LULS42 cells produced higher viral titers and exhibited faster replication kinetics compared to the other cell lines, including *H. anatolicum* (HAE/CTVM9) cells previously reported as a model for the study of CCHFV and HAZV replication [16,34,35]. These findings highlight potential biological differences among tick species that may influence viral replication efficiency.

Following the characterization of infection dynamics, we verified the presence of vDNAs also in *H. lusitanicum* HLE/LULS42 tick cells and investigated the role of vDNAs in the establishment of persistent infections. Our results demonstrate that HAZV establishes a persistent, non-cytopathic infection in tick cells, as indicated by the lack of cell death and the absence of significant morphological changes. These findings align with previous studies involving other tick-borne viruses, such as CCHFV, where the virus persists in tick cells without inducing cytopathic effects [16,34].

Using a panel of primers targeting the S segment of the HAZV genome [16], we successfully detected vDNAs in infected HLE/LULS42 cells. vDNAs were present across different MOIs (0.1 and 1), and their presence persisted over 60 days post-infection, indicating that vDNAs formation is a sustained feature of viral infection in these cells. Interestingly, vDNAs were detected in both the nucleus and the cytoplasm, further supporting the hypothesis of retrotransposons involvement and suggesting potential distinct functional roles in different cellular compartments. This contrasts with studies in mosquitoes, where vDNAs have been found predominantly in the cytoplasm, but usually at early time points after infection [17].

Our findings align with previous work in this field, particularly studies demonstrating that vDNAs plays a key role in regulating viral replication and promoting tick cell survival during infection [16]. To further confirm this, we employed AZT, a reverse transcriptase inhibitor, to inhibit vDNAs formation. AZT treatment resulted in a significant reduction in vDNAs production, which correlated with decreased cell viability, while viral genome replication and viral progeny production remained unaffected. These results strongly suggest that vDNAs are not merely a byproduct of viral infection but actively modulate the tick cell environment to reduce viral cytotoxicity.

A notable discrepancy between our findings and those of a previously referenced study [16] was the observed viral titers in infected cells treated with AZT. While the referenced study used an immunofluorescence assay to measure infectious virus particles, our study did rely on RT-qPCR for viral genome quantification, which could account for the observed differences. In fact, results by RT-qPCR can be affected by viral RNA released by death cells and not correlate with the number of infectious viral particles. Nevertheless, both studies support the hypothesis that vDNAs functions as a regulatory mechanism that modulates viral replication to maintain host cell survival. Interestingly, our results indicate that AZT-treated cells produced a higher number of viral particles per cell, suggesting that vDNAs may serve as a regulatory checkpoint that limits viral replication to levels compatible with host cell survival. This aligns with the hypothesis that vDNAs contributes to RNA interference (RNAi)-mediated antiviral responses, particularly in the generation of small interfering RNAs (siRNAs) that target viral RNA for degradation. The RNAi pathway is a well-established antiviral mechanism in arthropods, playing a central role in limiting viral replication and enabling the host to tolerate chronic infections [16,17,21,23,24,36,37]. In the case of vDNAs, it is plausible that these retro-transcribed DNA molecules serve as templates for siRNA production, which in turn regulates viral replication. Previous studies in mosquitoes and *Drosophila* have shown that RNAi-mediated responses are crucial for controlling viral infections, and vDNAs enhances the effectiveness of this pathway [23,24]. However, integrating approaches like small RNA-seq or other next-generation sequencing techniques to map siRNAs derived from vDNAs can provide valuable insights into the role of vDNAs in RNAi-mediated antiviral responses in tick cells. Our findings suggest that a similar mechanism may be at play in tick cells infected with HAZV, where vDNAs modulates the host immune response to ensure viral persistence without inducing cell death.

One of the most significant findings of this study, consistent with prior discoveries [16], is that vDNAs is not a stable entity and requires continuous synthesis to maintain its function. This is supported by the significant reduction in vDNAs detection following AZT treatment. The transient nature of vDNAs formation suggests that these molecules exist predominantly in episomal form rather than being integrated into the tick genome. Episomal vDNAs likely acts as intermediate regulatory molecules, interacting with host cellular machinery to balance viral replication and immune responses.

By limiting viral replication and enhancing tick cell survival, vDNAs contributes to the establishment of persistent infections, ensuring the virus remains within the tick population and facilitating its transmission to vertebrate hosts. Future research should aim to identify the host factors involved in vDNAs biogenesis, determine whether similar mechanisms occur in other arthropod vectors, and explore the implications of these findings for the control of tick-borne viruses. Moreover, validating these findings in the context of CCHFV and persistently infected living ticks is essential, as experimental models, though invaluable, cannot fully replicate the complexity of natural infection. Furthermore, studying other nairoviruses and examining these processes within the same framework is critical for gaining a comprehensive understanding of virus–vector interactions.

Understanding these interactions at the molecular level may ultimately provide new targets for antiviral strategies, improving our ability to mitigate the public health risks posed by tick-borne viruses.

## 5. Conclusions

In conclusion, this study provides strong evidence that vDNAs plays a central role in the establishment and maintenance of persistent viral infections in tick cells. By regulating viral replication and promoting host cell survival, vDNAs enables ticks to tolerate chronic infections, which is crucial for their role as long-term virus reservoirs. The dynamic nature of vDNAs synthesis, coupled with their ability to modulate the RNAi-mediated antiviral response, highlights their importance in the coevolutionary relationship between arboviruses and their tick vectors. Future research should focus on elucidating the precise mechanisms through which vDNAs modulate viral replication and host immune responses, as well as validating these findings in live tick models. Ultimately, a deeper understanding of vDNAs-mediated persistence could lead to novel strategies for controlling tick-borne viral diseases.

## Figures and Tables

**Table 1:** Sequences of primer and probe used for the detection of HAZV.

Primer/Probe HAZV	Sequence 5′→3′
Primer forward	TGCCGAAATTCCTCAGCTCGAC
Primer reverse	TGCACACTCCATGATAGGAGCAC
Probe	FAM-AGGGACGCCATCTACAGCTCAGCACTCA-BHQ1

**Table 2:** Cycling conditions of the RT-qPCR.

	Temperature	Time	Cycles
Initial denaturation	95 °C	5 min	1
Denaturation	95 °C	10 s	
Annealing	60 °C	30 s	40
Extension	72 °C	20 s	

**Table 3:** Cycling conditions of the conventional PCR.

	Temperature	Time	Cycles
Initial denaturation	95 °C	5 min	1
Denaturation	95 °C	15 s	
Annealing	60 °C	30 s	45
Extension	72 °C	20 s	
Final extension	72 °C	5 min	1

**Table 4:** Efficiency and R values calculated for several probe concentrations.

Probe nM	Primer 250 nM		Primer 400 nM	
	Efficiency	R	Efficiency	R
50	82.30%	0.986	71.00%	0.993
100	78.00%	0.997	83.50%	0.966
150	81.20%	0.992	76.40%	1.0
200	74.00%	0.996	81.40%	1.0
250	82.70%	0.991	94.30%	0.979

Note: Efficiency represents the ratio of amplified target molecules per PCR cycle, with an optimal range between 90% and 110%. The correlation coefficient (R) evaluates how well the generated data fit the standard curve, with values > 0.990 considered indicative of a strong linear relationship.

## Data Availability

All data generated in this study are available within this article.

## Abbreviations

The following abbreviations are used in this manuscript:

RT-qPCR	Quantitative reverse transcription polymerase chain reaction
DNA	Deoxyribonucleic acid
RNA	Ribonucleic acid
MOI	Multiplicity of infection
